# Editorial: Public Health Nutrition in the Middle East

**DOI:** 10.3389/fpubh.2016.00033

**Published:** 2016-03-01

**Authors:** Nahla Hwalla, Connie M. Weaver, Rania A. Mekary, Sibelle El Labban

**Affiliations:** ^1^American University of Beirut, Beirut, Lebanon; ^2^Purdue University, West Lafayette, IN, USA; ^3^MCPHS University, Boston, MA, USA

**Keywords:** public health nutrition, Middle East, nutrition transition, obesity, undernutrition, capacity building, policy, interventions

**The Editorial on the Research Topic**

**Public Health Nutrition in the Middle East**

## Public Health Nutrition in the Middle East: Situation Analysis

The Middle East is one region in desperate need for public health nutritionists and public health nutrition strategies to address the ever escalating burden of diet-related diseases. The Middle Eastern region has been witnessing a triple burden of disease, characterized by the simultaneous presence of undernutrition, micronutrient deficiencies, and overweight and obesity as a consequence of both emergency situations and nutrition transition that are placing different communities in the region at various nutrition-related health risks (1–6). In fact, recent sociodemographic changes resulting from the unstable political crisis in some countries have placed refugees, internally displaced individuals, and other socially and politically vulnerable populations at high risk of food and nutrition insecurity, as well as undernutrition and its comorbidities (1, 2). In parallel, populations of the region have been experiencing a rapid nutrition transition characterized by a shift away from a traditional, more seasonal, and more diverse diet, rich in whole grains, fruits, and vegetables, towards a “westernized” diet that is rich in refined carbohydrates, animal protein, total, saturated, and trans fats, sugar, and salt. In fact, data from the Food and Agriculture Organization food balance sheets and from food consumption surveys in the region have highlighted a shift towards an increasingly energy-dense diet and high intake of fat and sugar, coupled with a parallel decrease in complex carbohydrate consumption (7–13). Factors driving this transition include economic growth and increased incomes, globalization of trade and marketing, as well as rapid urbanization (5). These factors have further promoted lifestyle changes and encouraged sedentary behavior, particularly among adolescents (11–17 years) in the Eastern Mediterranean Region who showed the highest levels of insufficient physical activity (87.5%) in 2010 as compared to other regions (14). The aforementioned interrelated socio-economic, behavioral, environmental, and dietary determinants may have contributed to a drastic increase in rates of chronic non-communicable diseases, such as obesity (5), diabetes (4), cardiovascular diseases (3), and cancer (6). While several countries of the region have obesity rates exceeding 30%, rates of undernutrition, particularly stunting, among under-five children in low- and middle-income countries remain high (15).

To tackle the complex nature and multi-faceted aspect of malnutrition that is burdening the region, effective culturally sensitive nutrition programs and policies must be developed and implemented. Capacity building to draft and implement such policies is also crucial for success. This can be achieved through a solid practice and framework of public health nutrition to effectively address, through both prevention and treatment, the triple burden of disease in the Middle East. Most graduates of this field are dietetic practitioners and nutritionists.

## Significance of Public Health Nutrition in the Middle East

Public health nutrition is a multidisciplinary approach that combines the disciplines of public health and the science of nutrition, and it is more colloquially defined as “nutrition for the public.” The fact that public health nutrition aims to address lifestyle- and nutrition-related health problems that ail countries of the region by promoting and safeguarding the nutritional health and well-being of communities and populations makes it a highly demanded and attractive field in the Middle East.

Existing shortfalls in public health nutrition interventions in the Middle East have led to the lack of human resources and evidence-based policies (16). This partly stems from limited nutritional surveillance data, a focus on curative rather than preventive measures for nutrition-related health problems, and limited operational research on effectiveness of population-wide interventions (16, 17). These issues are compounded by a regional focus on individual consultations and clinical practice as depicted by a plethora of dietitians in hospitals and clinics that intervene on a one-on-one basis rather than on a community level. Moreover, despite the abundance of public health professionals and nutritionists/dietitians in the region, their practice to date remains tied to one of either disciplines; hence, their independent impact on the public health remains less remarkable, whereas the situation analysis of the region calls for professionals who blend the skills and qualifications of both professions, i.e., public health and nutritional sciences. Consequently, instilling public health nutrition by graduating, training, recruiting, and empowering public health nutritionists remains more potent and synergistically beneficial for public health of the populations in the Middle East.

Certified public health nutritionists will be well-equipped with up-to-date knowledge and applied functional skills in one or more specialty areas, such as research, policy development, program planning, health promotion and education, monitoring, and evaluation. Their primary role consists of dedication and commitment to better assist organizations and communities at large in developing and implementing evidence-based nutrition programs and interventions at local, national, and regional levels.

## Capacity Building for Public Health Nutrition in the Middle East

Countries in the Middle East are in need of human resources to develop, implement, evaluate, and set policies to tackle the triple burden of disease ailing these countries. Academic institutions can provide evidence-based knowledge needed for the design, implementation, monitoring, and evaluation of such programs. Research findings will inform policy makers for action. Universities should consider developing curricula for programs in the field of public health nutrition, which are lacking in the region.

On the other hand, capacity building should foster accountability, whereby evaluation should be incorporated in the implementation of nutrition programs. Continuous monitoring and reporting of updated data should be emphasized as a way to follow-up and ensure the implementation of every step in the program. Nutritional surveillance systems in countries of the region are fundamental for monitoring nutrition interventions and assessing nutritional status, food availability and consumption, and physical activity patterns of populations. The impact of programs and policies aimed at reducing the burden of food- and nutrition-related diseases should be also constantly assessed and evaluated. Lead agencies must be made accountable.

## Conclusion

In conclusion, public health nutrition is largely neglected in the Middle East in terms of human resources and public policies that are fundamental to address diet-related problems in countries of the region. There is an urgent need to address the triple burden of disease in the Middle East through nutrition policies and preventive programs grounded in scientific evidence. Proficient and motivated public health nutritionists who are equipped with the necessary skills and qualifications are essential to respond to this need, along with a multisectoral commitment that builds on effective and long-term capacity building.

The aim of this Research Topic is to cover, to an appreciable extent, different aspects of public health nutrition in the Middle East that draw attention to most crucial needs, challenges, caveats, and lessons learned in this field and which call for future solutions and initiatives.

## Author Contributions

NH, CW, RM, and SEL have equally contributed to the content of this work and fulfilled the authorship criteria as listed by Frontiers.

## Conflict of Interest Statement

The authors declare that the research was conducted in the absence of any commercial or financial relationships that could be construed as a potential conflict of interest.
